# miR-203 inhibits proliferation and self-renewal of leukemia stem cells by targeting survivin and Bmi-1

**DOI:** 10.1038/srep19995

**Published:** 2016-02-05

**Authors:** Yi Zhang, Shu-yan Zhou, Hai-zhao Yan, Dan-dan Xu, Hai-xuan Chen, Xiao-yan Wang, Xiao Wang, Yu-ting Liu, Li Zhang, Sheng Wang, Peng-jun Zhou, Wu-yu Fu, Bi-bo Ruan, Dong-lei Ma, Ying Wang, Qiu-ying Liu, Zhe Ren, Zhong Liu, Rong Zhang, Yi-fei Wang

**Affiliations:** 1 College of life science and technology, Jinan University, Guangzhou, 510632, P.R. China; 2 Institute of Biomedicine, Jinan University, Guangzhou, 510632, P.R. China; 3 Department of Pathological Physiology, Wan-nan Medical College, Wuhu, 241000, P.R. China; 4 College of medicine, Jinan University, Guangzhou, 510632, P.R. China; 5 Department of Pathogen Biology and Immunology, Medical College, Guangdong Pharmaceutical University, Guangzhou 510006, China; 6 Department of Endoscopy, Sun Yat-sen University Cancer Center, State Key Laboratory of Oncology in South China,Collaborative Innovation Center for Cancer Medicine, Guangzhou, 510632, P.R. China; 7 Section of Otolaryngology, Department of Surgery, Yale School of Medicine, U.S.A

## Abstract

Drug resistance is one of the leading causes of failed cancer therapy in the treatment of acute myeloid leukemia. Although the mechanisms of resistance are poorly understood, they may be related to the presence of leukemia stem cells (LSCs). Down-regulation of the miR-203 reportedly contributes to oncogenesis and chemo-resistance in multiple cancers. We found that miR-203 expression was down-regulated in CD34 + AML cells as compared with CD34− cells isolated from patients as well as in LSC-enriched (CD34 + CD38−) cell lines KG-1a or MOLM13. Additionally, re-expression of miR-203 led to decreased cell proliferation, self-renewal, and sphere formation in LSCs. Moreover, miR-203 was found to directly target the 3′un-translated regions of survivin and Bmi-1 mRNAs affecting proliferation and self-renewal in LSCs. In this study, we identified a novel miR-203/survivin/Bmi-1 axis involved in the regulation of biological properties of LSCs. This axis may represent a new therapeutic target for acute myeloid leukemia and a potential prognosis/diagnostic marker for LSCs therapy.

Acute myeloid leukemia (AML) is one of the most common hematological malignancies in adolescents and young adults^1^. Despite substantially improved survival rates over recent decades, many AML patients still die due to drug resistance following treatment. Although the mechanisms for drug resistance are likely to be complex, one possible cause is the presence of cancer stem cells (CSCs) or tumor initiation cells, which participate in tumor progression, metastasis, and recurrence^2^. Leukemia stem cells (LSCs) were first identified by J. E. Dick (CD34 + CD38− cells) and subsequently validated by numerous researchers^3^,^4^. A small proportion of these cells are capable of escaping from the immune system surveillance, surviving under stress conditions and re-initiating tumor development, which may result in relapse, drug resistance, or even death^5^.

microRNAs (miRNAs) comprise a class of small non-coding RNAs of 19–22 nucleotides, which target mRNAs by binding to their 3′un-translated regions (3′-UTR) and inducing mRNA degradation or translational repression in a manner dependent on sequence complementarity^6^. Accumulating evidence suggests that miRNAs are frequently deregulated in various malignancies, and that they function as oncogenes (e.g., miR-21 and miR-17–29) or tumor suppressors (e.g., miR-34 and let-7) by regulating multiple cellular functions, including proliferation, senescence, apoptosis, and chemo-resistance^7^,^8^,^9^,^10^. In recent years, miRNA profiling and characterization of their targets have been reported for various forms of leukemia, including AML^11^, chronic myeloid leukemia^12^, T lymphocytic leukemia^13^, Blymphocytic leukemia^14^, and multiple myeloma^15^. These studies have revealed an important link between expression of miRNAs and development of hematological diseases.

Several studies have reported that miR-203 functions as a tumor suppressor in various cancers, including leukemia, esophageal cancer, and breast cancer^16^,^17^,^18^, inhibiting cancer cell proliferation. Taipaleenmaki *et al.* showed that miR-203 is down-regulated in primary breast cancer and that its re-expression significantly reduces tumor growth and metastases to the bone in nude mice^19^. Saini *et al.* reported that ectopic miR-203 attenuates the development and metastasis of prostate cancer^20^. Intriguingly, miR-203 has also been shown to regulate “stemness” through its target genes^21^. For example, Ju *et al.* reported that miR-203 suppression is essential for maintenance of stemness in colon cancer cells^22^. Taken together, these findings indicate that miR-203 could be a key repressor of proliferation and stemness in carcinogenesis. However, the role of miR-203 in the regulation of LSC functions remains to be elucidated.

In our previous work, we used magnetic sorting to enrich LSCs from AML cell lines KG-1a and MOLM13^23^. In the present study, we used the same approach to investigate biological functions of miR-203 in LSCs with the aim to identify the molecular mechanisms of its action. We report that miR-203 plays a pivotal role in maintaining proliferation and self-renewal abilities of LSCs through targeting canonical molecules such as survivin and Bmi-1. Moreover, we suggest that the miR-203/survivin/Bmi-1 axis could provide a potential therapeutic target for leukemia treatment.

## Results

### Down-regulation of miR-203 in LSCs is essential for their proliferation and self-renewal *in vitro*

**Figure 1:**
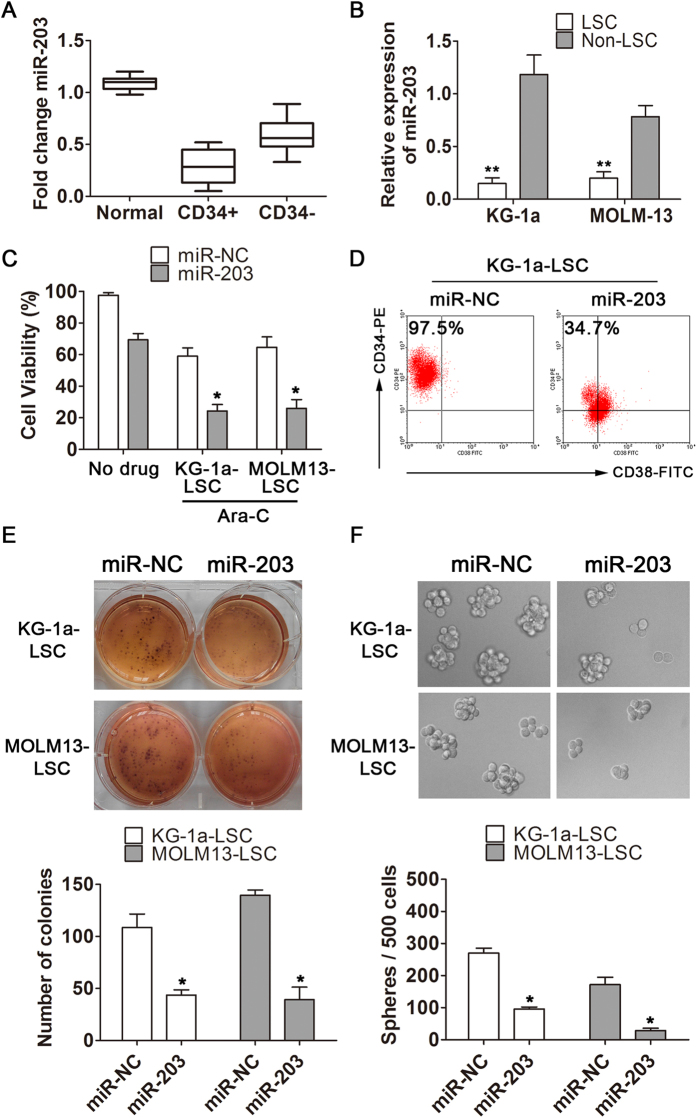
miR-203 is down-regulated in LSCs, and its over-expression inhibits proliferation and stemness *in vitro*. (**A**) qRT-PCR analysis of miR-203 expression was conducted on 15 peripheral blood samples from healthy volunteers and 50 paired CD34 + /CD34− cells from the blood samples of AML patients. (**B**) miR-203 expression was detected in LSC-enriched KG-1a and MOLM-13 cell lines and referenced against RNU6B. (**C**) Viability of KG-1a-LSCs and MOLM13-LSCs was analyzed following transfection with miR-NC or miR-203 with or without Ara-C. (**D**) In KG-1a-LSCs, the percentage of the CD34 + CD38− cells (upper left quadrant) in miR-NC and miR-203-transfected cells was detected by flow cytometry. (**E**) LSC cells transfected with miR-203 or miR-NC (48 h) were seeded in 6-well plates in soft agar at clonal density and cultured for 2 weeks. Cell colonies were then stained with Giemsa and photographed. (**F**) Representative photographs showing the spheroids formed by miR-203 or miR-NC expression in LSC cells. All data represent the mean ± SD from three independent experiments in each condition (*P < 0.05, **P < 0.01).

Several previous studies have demonstrated that miR-203 is down-regulated in several cancers, and that this promotes the progression and development of the disease^24^,^25^. To investigate whether miR-203 is down-regulated in leukemia and regulates biological functions of LSCs, we isolated CD34 + /CD34− cells from blood samples of 50 leukemia patients and collected 15 blood specimens from healthy subjects. In the next step, we measured miR-203 levels in these cell fractions and normal blood using quantitative RT-PCR (qRT-PCR). miR-203 was significantly down-regulated in CD34 + cells as compared to CD34− cells and normal blood (Fig. 1A). Concurrently, expression of miR-203 was lower in LSC-enriched cell lines KG-1a and MOLM13 relative to RNU6B (*P* < 0.01; Fig. 1B). To evaluate biological significance of miR-203 down-regulation, LSCs isolated from KG-1a and MOLM13 were transfected with miR-NC or miR-203, and cell viability analyzed with or without cytarabine (Ara-C), a small molecule compound used for AML treatment. Cell transfected with 50-nM miR-203 markedly increased miR-203 levels (Additional Figure S1). Intriguingly, miR-203 reconstitution reduced the viability of LSC-enriched cells compared with miR-NC transfected, and this effect was more pronounced when cells were additionally exposed to 2-μM Ara-C 48h after transfection with miR-203 (*P* < 0.05; Fig. 1C).

We then asked if miR-203 suppresses proliferation of LSCs affecting the proportion of CD34 + CD38− cells. To test this hypothesis, we used CD34-PE and CD38-FITC antibodies to stain KG-1a-LSCs after transfection with miR-203 or miR-NC and performed flow cytometry analysis. As shown in Fig. 1D, the percentage of LSCs (CD34 + CD38−) in miR-203-transfected cells (34.7%) was significantly less than in NC-transfected cells (97.5%). Hence, miR-203 reconstitution appeared to effectively suppress LSCs.

The ability to form spheroids in serum-free medium supplied with growth factors is a characteristic property of cancer stem cells. To determine the effects of miR-203 over-expression on LSC self-renewal and survival, we used spheroidogenesis and soft agar colony formation assays. miR-203 reintroduction reduced the number of clones in both LSC-enriched cell types compared with the clones observed in the miR-NC-transfection cells (*P* < 0.05; Fig. 1E). Similarly, the number and average diameter of spheroids in the miR-203-transfection cells were significantly lower than miR-NC-transfected cells (*P* < 0.05; Fig. 1F). These findings suggest that miR-203 suppresses LSCs the proliferation and self-renewal.

### Survivin is a direct target of miR-203

**Figure 2:**
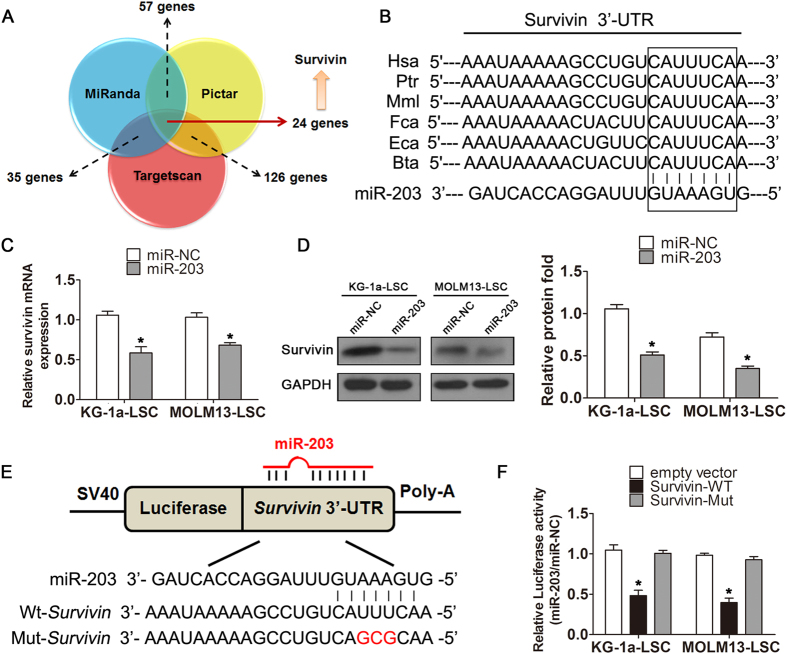
Survivin is a direct target of miR-203. (**A**) Venn diagram showing overlap in miR-203 targets predicted by three miR- research databases. The overlap contains 24 candidate genes that need further confirmation. (**B**) miR-203 directly targets the survivin 3′-UTR and is highly evolutionary conserved in different species (Has, Ptr, Mml, Fca, Eca, and Bta). (**C**) Survivin mRNA and (**D**) protein levels in miR-NC and miR-203 groups, analyzed by qRT-PCR and Western blot 48 h after transfection. GAPDH was used as an internal control (*P < 0.05). (**E**) Schematic diagram of the survivin3′-UTR pMIR-REPORT constructs and alignment of between mature wild-type (WT) and mutated (Mut) miR-203 putative target sites in the 3′-UTR of survivin. (F) LSC cells were co-transfected with pMIR-REPORT constructs containing empty (Ctrl), wild-type, or mutant target site of the survivin 3′-UTR plus miR-203 or miR-NC. Luciferase activity was normalized to *Renilla* activity and presented as relative to miR-NC (*P < 0.05). All graphs depict are representative of at least three independent experiments (mean ± SD).

Our previous work demonstrated that survivin is highly expressed in LSCs, and that it participates in drug resistance and anti-apoptotic functions^23^. Therefore, to investigate whether miR-203 regulates biological properties of LSCs through survivin, we used three miRNA databases (TargetScan, miRanda, and PicTar) to predict miR-203 binding sites in human mRNA transcripts. This analysis, identified 24 common genes that possessed miR-203 binding sites similar to the one from survivin 3′-UTR (Fig. 2A). Moreover, analysis of interspecies variation demonstrated that the miR-203 binding site in the 3′-UTR sequence of surviving is evolutionary conversed (Fig. 2B). To assess whether over-expression of miR-203 had any influence on survivin levels, we examined survivin mRNA and protein expression, 24 h after transfection, in both LSC-enriched cell lines. As shown in Fig. 2C, survivin mRNA levels were significantly reduced miR-203 transfection compared with miR-NC (*P* < 0.05). Correspondingly, miR-203 over-expression also reduced survivin protein expression (Fig. 2D). To investigate whether miR-203 directly targetes the survivin 3′-UTR sequence, we cloned the survivin 3′-UTR (containing the predicted miR-203 binding site) into the pMIR-REPORT luciferase reporter vector (Fig. 2E). Cells transfected with the pMIR-REPORT-survivin wild-type 3′-UTR vector and miR-203 exhibited a sharp reduction in luciferase activity in both KG-1a-LSCs and MOLM13-LSCs relative to cells transfected with the β-actin-*Renilla* luciferase plasmid, which was used as a control (*P* < 0.05; Fig. 2F). In contrast, luciferase activity remained unchanged when a mutant survivin 3′-UTR region (containing a mutation in the miR-203 binding site) was used, which provides additional evidence that survivin is a direct miR-203 target.

### The role of miR-203-mediated survivin down-regulation in the proliferation and apoptosis of LSCs

**Figure 3:**
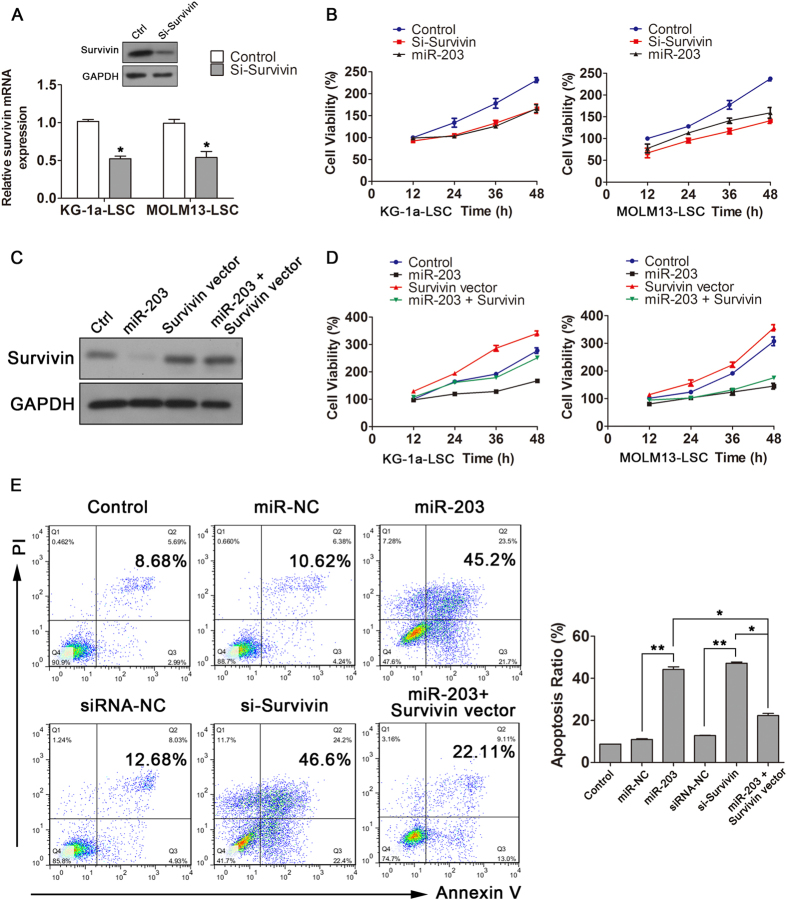
miR-203 inhibited proliferation and prompted apoptosis in LSCs. (**A**) Analysis of survivin mRNA and protein expression in KG-1a and MOLM13-LSCs 48 h after transfection with survivin-siRNA. Non-targeting (NT)-siRNA was used as a negative control (*P < 0.05). (**B**) The viability of LSCs was detected from 12 h to 48 h after transfection with survivin-siRNA or miR-203. (**C**) Analysis of survivin protein expression and (**D**) cell viability in LSCs following transfection with miR-203 and a survivin vector lacking the survivin 3′-UTR. (**E**) Analysis of apoptosis rates in KG-1a-LSCs 48 h after transfection with miR-203, survivin-siRNA, and survivin transgene devoid of its 3′-UTR. miR-NC and siRNA-NC were used as negative controls (^*^P < 0.05, ^**^P < 0.01).

To investigate whether miR-203 down-regulates survivin, two LSC-enriched cell types (KG-1a-LSCs and MOLM13-LSCs) were transfected with miR-203 and siRNA against survivin to determine cell proliferation and apoptosis rate. As shown in Fig. 3A, anti-survivin siRNA reduced survivin expression at both mRNA and protein levels as compared with negative control. Furthermore, cells transfected with si-survivin or miR-203 showed a significantly lower proliferation rate compared with controls (as measured with a Cell Counting Kit-8 (CCK-8) assay; Fig. 3B). We also investigated whether over-expression of survivin could reverse the inhibitory effect of miR-203 on LSCs proliferation. Specifically, we performed “rescue” experiments by co-transfecting LSCs with an miR-203 mimic and survivin-overexpressing construct that contained survivin cDNA devoid of its 3′-UTR (which could not be targeted or inhibited by miR-203) (Fig. 3C). In this experiment, in both cell lines survivin-overexpressing cells transfected with miR-203 proliferated more effectively than cells transfected with miR-203 only (Fig. 3D).

Additionally, KG-1a-LSCs transfected with miR-203, miR-NC, si-survivin, or siRNA-NC were analyzed 48 h post transfection for apoptosis by flow cytometry (Fig. 3E). The percentage of apoptotic cells was markedly higher in the miR-203 transfected cells than in the control group (45.2% vs. 10.62%, respectively; *P* < 0.01). Moreover, transfection with si-survivin also significantly increased the apoptosis rates relative to those in the siRNA-NC-transfected cells (46.6% vs. 12.68%, respectively; *P* < 0.01). However, the apoptosis rate of cells co-transfected with miR-203 and survivin construct devoid of the 3′-UTR (22.11%), was lower than that of miR-203 and si-survivin transfected cells. Collectively, these results suggest that miR-203 inhibits LSC proliferation and promotes apoptosis by targeting surviving via its 3′-UTR.

### miR-203 targets several stem cell regulatory factors

**Figure 4:**
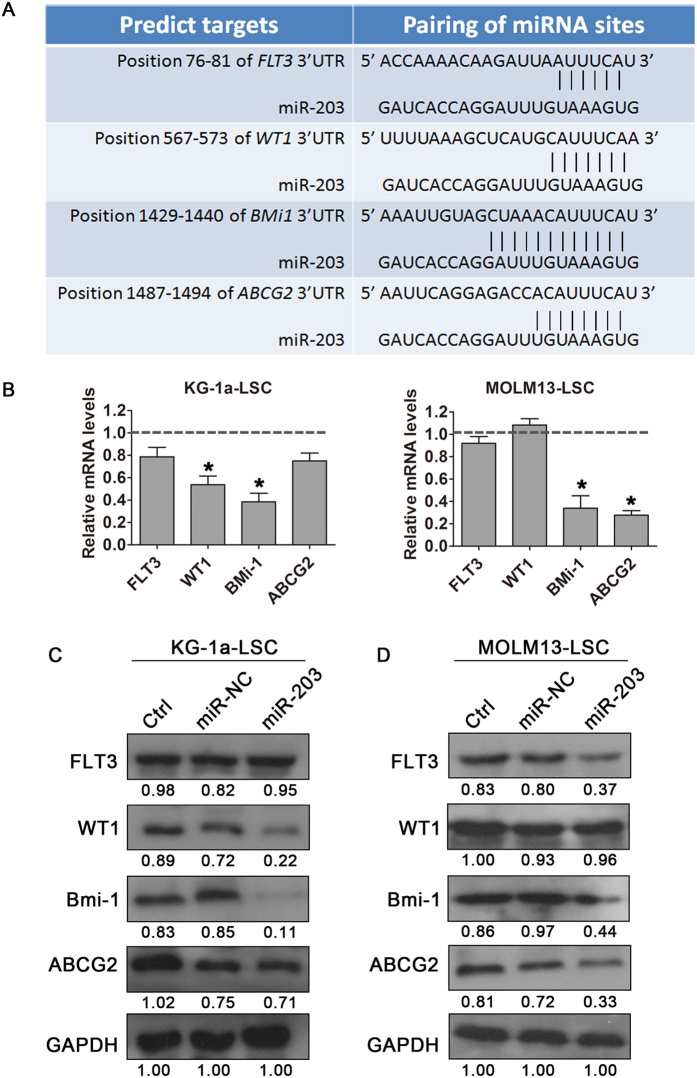
miR-203 regulates several self-renewal genes in LSCs. (**A**) Predicted binding targets of miR-203 to the 3′-UTRsof FLT3, WT1, Bmi-1, and ABCG2. (**B**) qRT-PCR analysis of miR-203 effects on the mRNA levels of candidate genes in LSCs. GAPDH was used as an internal control and data shown relative to the effects of miR-NC (*P < 0.05). (**C**) Western blot analysis of miR-203 effects on the protein levels of FLT3, WT1, Bmi-1, and ABCG2 in LSC cells. Densitometric values relative to miR-NC cells are provided. GAPDH was used as a loading control.

To further elucidate the mechanisms through which miR-203 regulates the stemness of LSCs (Fig. 1E), we sought additional potential miR-203 targets usingmiRanda, TargetScan, and PicTar. From these research databases, we selected four genes of interest, FLT-3, WT1, Bmi-1, and ABCG2 (Fig. 4A), known as oncogenic and stem cell regulators that participate in leukemia initiation and progression. We observed that miR-203 over-expression reduced mRNA levels of these genes in a cell type-dependent manner: in KG-1a-LSCs, miR-203 reduced WT1 and Bmi-1 levels, while in MOLM13-LSCs miR-203 selectively reduced levels of Bmi-1 and ABCG2 (GAPDH was used as an internal control; Fig. 4B). Western blotting analysis of FLT-3, WT1, Bmi-1, and ABCG2 protein levels was consistent with the results of qRT-PCR. Interestingly, miR-203 over-expression reduced Bmi-1 protein levels in both LSC-enriched cell lines with a decrease from 50% to almost 100% compared with NC-transfected cells (Fig. 4C). Moreover, the miR-203 effects on the FLT-3, WT1, and ABCG2 protein levels differed in two cell lines suggesting that miR-203 involvement is cell type-dependent.

### Bmi-1 is a direct and functional target of miR-203 in LSCs

**Figure 5:**
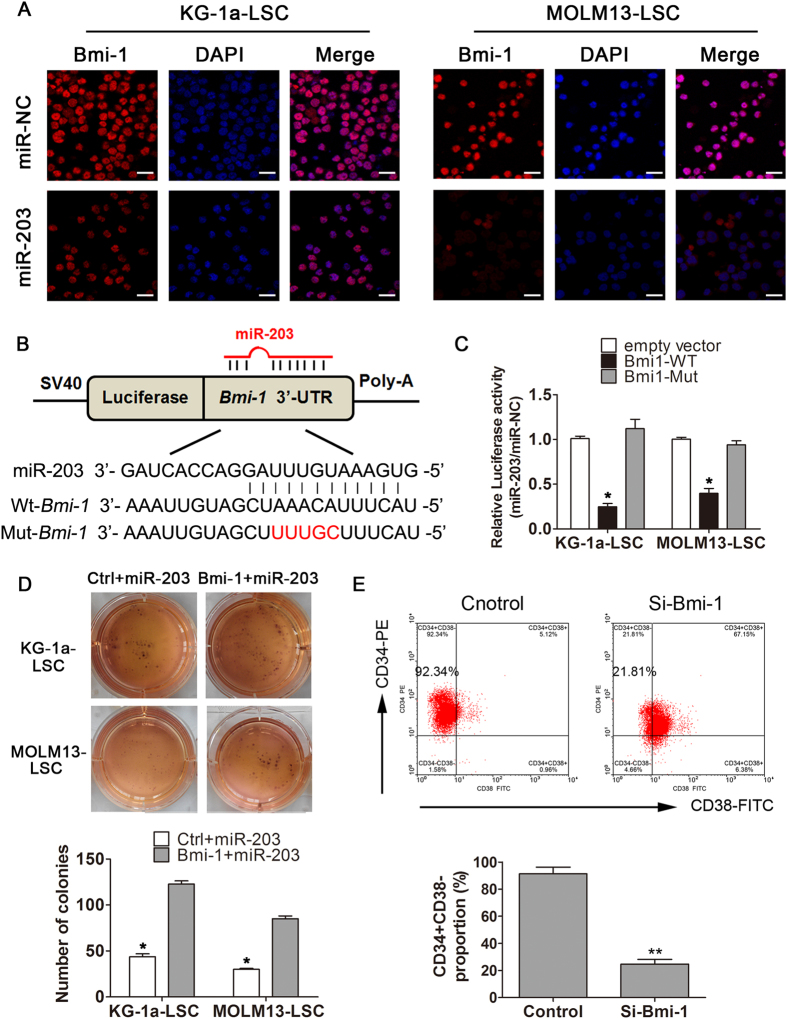
Bmi-1 is a direct target of miR-203. (**A**) KG-1a and MOLM13-LSCs were transfected with miR-NC or a miR-203 mimic (50 nmol/L; 48 h) and cultured on slides overnight following immune-staining. (**B**) Schematic description of the hypothesized interactions between miR-203 and the wild-type (WT) or mutant (Mut) putative target regions in the 3′-UTR of Bmi-1. (**C**) LSC cells were co-transfected with pMIR-REPORT constructs containing wild-type, or mutant miR-203 target sites in the Bmi-1 3′-UTR or with empty vector(Ctrl) plus miR-203 or miR-NC. Luciferase activity was normalized to *Renilla* and presented as relative to miR-NC (*P < 0.05). (**D**) LSCs were co-transfected with Bmi-1 cDNA vector devoid of the Bmi-1 3′-UTR and miR-203 and used in a soft agar colony formation assay (*P < 0.05). (E) In KG-1a-LSCs, the percentage of the CD34 + CD38− cells (upperleft quadrant) in the control and si-Bmi-1-transfected cells was detected using flow cytometry (**P < 0.01). All graphs depict representatives of at least three independent experiments (mean ± SD).

In both LSC-enriched cell lines, the Bmi-1 mRNA and protein levels were reduced by miR-203 (Fig. 4B,C). These data are consistent with previous reports that characterized Bmi-1 as a cancer stem cell regulator^26^,^27^. To explore whether Bmi-1 is a direct miR-203 target, we transfected LSCs with miR-203 or miR-NC, then used immuno-fluorescence to detect Bmi-1. As shown in Fig. 5A, compared with miR-NC, miR-203 significantly reduced cell growth and Bmi-1 expression levels in LSCs. Moreover, we cloned the Bmi-1 3′-UTR harboring the miR-203 binding site downstream of luciferase in firefly plasmid pMIR-REPORT (Fig. 5B). Co-transfection of KG-1a-LSCs and MOLM13-LSCs with this luciferase vector and miR-203 mimic led to decreased luciferase activity (Fig. 5C). Mutation of the miR-203 binding sequence in the Bmi-1 3′-UTR abolished the suppressive effects of miR-203, confirming that Bmi-1 is a direct miR-203target.

To investigate whether Bmi-1 is a functional target of miR-203, we also performed rescue experiments by co-transfecting KG-1a-LSCs and MOLM13-LSCs with a miR-203 mimic and Bmi-1 construct devoid of the 3′-UTR (and therefore protected from miR-203 targeting). As shown in Fig. 5D, in both cell lines Bmi-1 over-expression was sufficient to increase the number of clones grown in soft agar relative to co-transfection with miR-203 and a control vector (*P* < 0.05). Moreover, silencing of Bmi-1 with specific siRNA markedly reduced the proportion of LSCs from 92.34% to 21.81% (*P* < 0.01;Fig. 5E).

### Bmi-1 regulates the self-renewal ability of LSCs

**Figure 6:**
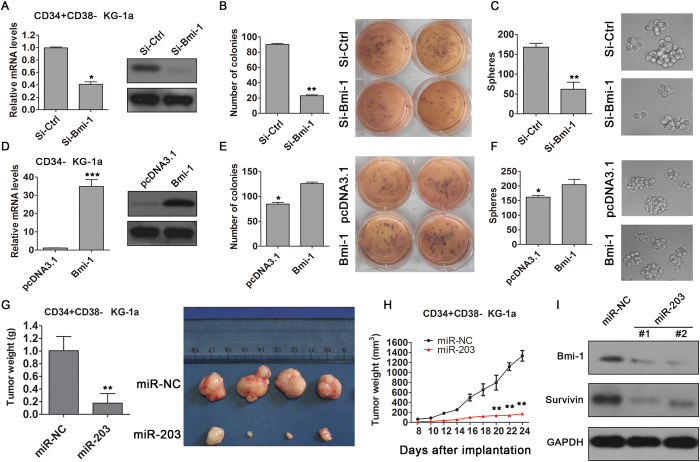
Effects of Bmi-1 on the self-renewal properties of LSCs and miR-203 suppressed tumor growth *in vivo*. (**A**) Validation by qRT-PCR and Western blot analyses of Bmi-1 siRNA-mediated knockdown effects in CD34 + CD38− cells purified from KG-1a. (**B**) Soft agar colony and (**C**) spheroid formation assays in anti-Bmi-1 siRNA-transfected CD34 + CD38− KG-1a cells. (**D**) Validation by qRT-PCR and Western analyses of Bmi-1 expression in CD34- KG-1a cells upon infection with pcDNA3.1-Bmi-1 construct encoding full-size Bmi-1 mRNA. pcDNA3.1 was used as a control. (**E**) Soft agar colony and (**F**) spheroid formation assays with CD34- KG-1a cells transfected with empty vectors and pcDNA3.1-Bmi-1 construct. All data are representative of three independent experiments (mean ± SD; ^*^P < 0.05, ^**^P < 0.01). (**G**) Xenograft tumors derived from control and miR-203-transfected KG-1a LSCs (1 × 10^5^ cells per mice, 4 animals for each group) were weighed and photographed immediately after tumor extraction(mean ± SD; ^**^P < 0.01). (**H**) Tumor volumes were measured with a Vernier caliper and their volumes calculated at indicated days (mean ± SD; ^**^P < 0.01). (I) Bmi-1 and survivin protein levels were assessed using Western blot performed with control or miR-203-transfected xenograft tumor tissue.

To assess whether Bmi-1 regulates stemness in LSCs, we manipulated the Bmi-1 expression vector in LSCs (CD34 + CD38− and CD34− KG-1a cells using siRNA. Anti Bmi-1 siRNA decreased Bmi-1 mRNA and protein levels in CD34 + CD38− KG-1a cells (Fig. 6A) and reduced clone numbers as well as spheroid formation (*P* < 0.01, Fig. 6B,C). In contrast, Bmi-1 over-expression elevated the Bmi-1 mRNA and protein levels in CD34− KG-1a cells (*P* < 0.001; Fig. 6D) and increased the numbers of clones and spheroids compared with the control (empty plasmid pcDNA3.1) (*P* < 0.05; Fig. 6E,F). Taken together, these data support the hypothesis that Bmi-1 is a direct and functional target of miR-203 that mediates LSCs stemness.

### miR-203 suppresses LSCs maintenance *in vivo*

Since our functional assays suggested that miR-203 is involved in the regulation of LSCs proliferation and self-renewal, we tested if miR-203 could have potential therapeutic effects on tumor growth *in vivo*. For this purpose, we injected control (miR-NC) and miR-203-transfected LSCs into the flanks of nude mice. Following 22 ~ 24-day observations and measurements, the mice were sacrificed and xenograft tumors harvested for analysis. As shown in Fig. 6G–H, in comparison with the control cells, treatment with miR-203 significant suppressed growth rates and weights of tumors inoculated with LSCs. Notably, miR-203 dramatically suppressed the Bmi-1and surviving protein levels in xenograft tumor tissue (Fig. 6I). Taken together, the results of these proof-of concept experiments demonstrated that miR-203-mediated key targets (survivin and Bmi-1) are pivotal for LSCs progression and that targeting this novel miR-203/survivin/Bmi-1 axis could be a potentially effective therapeutic strategy against AML.

## Discussion

Accumulating evidence suggests the existence of tumorigenic sub-population of cancer cells that exhibits stem-like features such as self-renewal and limitless proliferation^28^. These cancer stem cells (CSCs) are involved in tumor initiation and propagation and contribute to radio- and chemotherapy resistance^29^,^30^. Therefore, understanding of molecular mechanisms that drive CSCs is essential for development of innovative therapeutic interventions.

**Figure 7:**
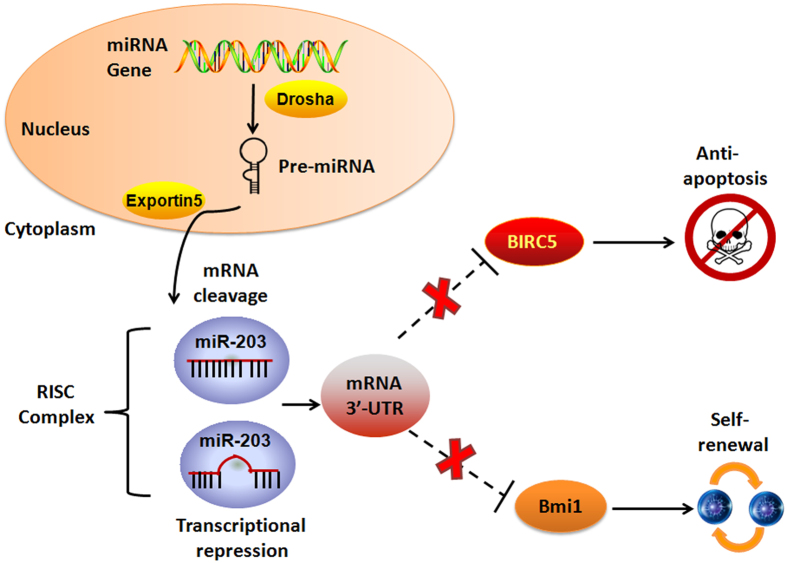
Schematic representation of the proposed miR-203 role in the regulation of LSCs proliferation and self-renewal. Mature miR-203 is cleaved and transported out of the nucleus where it exhibits effects on cell growth and self-renewal through several mechanisms. Loss of miR-203 results in over-expression of the anti-apoptosis protein BIRC5 (survivin), which promotes chemo-resistance in LSCs. In addition, miR-203 negatively regulates self-renewal of LSCs by targeting the Bmi-1 3′-UTR.

Dysregulation of miRNA expression has been correlated with various human diseases. Furthermore, many miRNAs participate in tumor initiation, progression, and metastasis by targeting mRNA expression and functioning as oncogenes or tumor suppressors in different conditions. We have previously reported that over-expression of an anti-apoptotic protein survivin contributes to drug resistance in LSCs^23^. However, the molecular mechanisms underlying higher expression of survivin in LSCs are likely to be complex. In the present study, we demonstrated that down-regulation of miR-203 leads to LSCs proliferation and self-renewal mediated via survivin and Bmi-1 expression. Moreover, knockdown of survivin and Bmi-1significantly reduced cell viability, induced apoptosis, and decreased spheroid formation in LSCs. Our rescue experiments indicated that miR-203 effects require its binding to the 3′-UTRs of survivin and Bmi-1. Additionally, miR-203 dramatically suppressed tumor growth *in vivo* when compared with control. Therefore, our study shows for the first time, that miR-203 is a critical regulator of proliferation, chemo-resistance, and maintenance of stemness in LSCs, and that these effects are mediated via survivin and Bmi-1 expression regulation (Fig. 7).

miR-203 was first identified as a key molecule that controls proliferation of keratinocytes^31^ and represses stemness to promote epithelial differentiation^32^. More recently, Wellner *et al.* demonstrated that miR-203 represses the stem cell genes Sox2 and Klf4 to inhibit tumorigenesis in pancreatic cancer^33^. In addition, Zhou *et al.* suggested that miR-203 inhibits cell growth and invasion by targeting CASK in gastric cancer^34^. Wang *et al.* reported that miR-203 acts as a tumor suppressor in lung cancer cells through inhibition of SRC translation^35^. In other studies, miR-203 was shown to be involved in the regulation inflammatory responses and alleviation of neuronal incision injury^36^,^37^. In our study, we expanded knowledge on miR-203 activities via characterization of its two key targets, survivin and Bmi-1, which play essential roles in leukemia.

Survivin, a key inhibitor of apoptosis, is frequently up-regulated in many human cancers but almost undetectable in normal cells or tissues. These properties raise interest to survivin as a promising target for cancer therapy. In the present study, we found that survivin is a direct miR-203 target involved in LSC proliferation. Specifically, transfection with miR-203 significantly reduced survivin expression, inhibited cell proliferation, and induced apoptosis in LSCs. Conversely, these suppressive miR-203 effects were rescued when survivin transgenes devoid of its 3′-UTR miR-203-binding site were used. However, these are initial findings and further investigation is required to fully appreciate the role of the miR-203/survivin axis in cell cycle-driven proliferation/mitosis.

Bmi-1(polycomb-group protein B-cell-specific Moloney murine leukemia virus integration site 1) has been associated with self-renewal of adult stem cells, and its silencing results in cell cycle arrest and senescence^38^. Bmi-1 is also required for maintenance of stemness in several cancers. Chiba *et al.* found that Bmi-1 plays an important role in the maintenance of tumor-initiation properties of side population cells isolated from hepatocellular carcinoma^39^. Jin *et al.* showed that suppression of Bmi-1 inhibited the tumorigenic potential of prostate cancer cells^40^. In our study, we demonstrated that Bmi-1 is a direct miR-203 target involved in self-renewal. Thus, our findings raise interest to Bmi-1 as a clinical target that could be used for designing anti-CSC therapies.

Notably, miR-203 has been well documented as a tumor suppressor because it negatively regulates multiple oncogenes such as Src, BCR/ABL, and survivin. However, the mechanisms of miR-203 down-regulation may be tissue and cancer-specific. In recent years, numerous studies have demonstrated that promoter hypermethylation leads to down-regulation of miR-203 in several cancer types. Diao *et al.* reported that hypermethylation of miR-203 is a frequent event in endometrial carcinomas, which is strongly associated with microsatellite instability^41^. Moreover, Huang *et al.* found that miR-203 was frequently down-regulated by promoter hypermethylation in rhabdomyosarcoma cell lines and clinical biopsies, and that re-expression of miR-203 achieved via DNA-demethylation agents inhibited cancer cell migration, proliferation, and promoted terminal myogenic differentiation^42^. In line with these reports, we observed in our preliminary studies on LSCs a partial methylation state of miR-203, and found out that 5-aza-2′-deoxycytidine (DNA-demethylation agents) significantly increased miR-203 expression (data not shown). Therefore, epigenetic regulation is likely to be involved in the miR-203 suppression that we observed in LSCs. Our future studies therefore will focus on the state of miR-203 promoter methylation and other potential epigenetic alterations.

In conclusion, we demonstrated that down-regulation of miR-203 in LSCs increases their proliferation and self-renewal capacities via targeting of survivin and Bmi-1. Hence, we identified a novel molecular mechanism beyond negative regulation of LSCs by a critical tumor-suppressing miRNA. Our results suggest that the miR-203/survivin/Bmi-1 axis in LSCs could be a valuable therapeutic target, and further studies focused on this axis may facilitate development of novel therapeutic strategies and potential diagnostic and prognostic markers for AML treatment.

## Materials and Methods

### Patient samples and ethics statement

Fresh blood samples were collected from 50 AML patients after obtaining informed consent from all participants. The study received approval from the Human Research Ethics Committee of the Overseas Chinese Hospital of Jinan University and Sun Yat-Sen University Cancer Hospital (Guangzhou, China). CD34 + /CD34− cells were purified by magnetic-activated cell sorting (MACS; Miltenyi Biotec) using Ficoll density gradient centrifugation and cultured in B27 media (1:50; Life Technologies, Carlsbad, CA, USA) supplemented with10 ng/mL basic fibroblast growth factor, and 20 ng/mL epidermal growth factor in serum-free DMEM/F12 media. Such methods were carried out in accordance with the approved guidelines. Blood samples from 15 healthy volunteers were used as a control. Clinical characteristics of patients that participated in this study are summarized in Supplemental Table S1.

### Quantification of miR-203 expression in LSCs

Total RNA was isolated using miRNA Isolation Kit (GenePharma, Shanghai, China). Levels of mature miR-203 were measured using TaqMan MicroRNA Assay according to the manufacturer’s instructions. Primers for miR-203 and endogenous control RNU6B were purchased from GenePharma. For qRT-PCR, the following conditions were applied while using Bio-Rad Light Cycler PCR system: 95 °C for 3min, followed by 40 cycles of 95 °C for 12 s and 62 °C for 40 s in a 10-μL reaction volume.

### Cell culture and transfection experiments

The leukemia cell lines KG-1a and MOLM13 were cultured inRPMI-1640 medium supplemented with 10% fetal bovine serum (Invitrogen, Carlsbad, CA). Subsequently, CD34 + CD38− subpopulations were separated from KG-1a and MOLM13 cells using magnetic sorting and cultured as previously described. The miR-203 mimic and non-targeting control, miR-NC, were purchased from GenePharma. Lipofectamine TM2000 (Invitrogen) was used to transfect LSCs with 50 nmol/L of the miR-203 mimic or miR-NC according to the manufacturer’s instructions. The first-line AML drug Ara-C was transfected into LSCs at the indicated concentrations. For survivin and Bmi-1 functional assays, anti-survivin siRNA and Bmi-1 siRNA were used to knockdown their respective expression. pMIR-REPORT-survivin 3′-UTR or pMIR-REPORT-Bmi-1 3′-UTR carrying a wild-type or mutant seed sequence for miR-203 were used to detect relative luciferase activities. pcDNA3.1-survivin and pcDNA3.1-Bmi-1 plasmids lacking the3′-UTR were co-transfected with a miR-203 mimic to conduct rescue experiments.

### RNA isolation and qRT-PCR

Total RNA was extracted from cultured cells using TRIzol reagent (Invitrogen) and reverse transcribed into cDNA according to manufacturer’s instructions. The mRNA expression levels of survivin, FLT3, WT1, Bmi-1, and ABCG2 in miR-203-transfected cells were evaluated with SsoFast EvaGreen Supermix (Bio-Rad, Hercules, CA, USA) by qRT-PCR. GAPDH was used as an internal control. All specific primers are listed in Supplemental Table S2.

### Western blot analysis

KG-1a-LSCs and MOLM13-LSCs were transfected with indicated amounts of miR-203 mimic, si-survivin, si-Bmi-1, pcDNA3.1-survivin, or pcDNA3.1-Bmi-1, andprotein levels were evaluated after 48 h. Cells were washed twice in ice-cold PBS, and lysed in RIPA buffer containing 1 mM PMSF with constant agitation. The protein lysates were separated by 10% SDS-PAGE, and transferred to polyvinylidene membranes (Millipore, USA). Subsequently, the polyvinylidene membranes were incubated with 1:1,000 diluted antibodies (all from Cell Signaling Technology) for survivin, FLT3, WT1, Bmi-1, ABCG2, and GAPDH. The protein bands were stained with enhanced chemiluminescence solution. Band intensities were normalized to GAPDH as a loading control.

### CCK-8 assay

For the CCK-8 assay, 5,000 cells were seeded into 96-well plates and transfected with various concentrations of drugs, plasmids, miR-203, and miR-NC for 48 h using Lipofectamine 2000. Subsequently, 10 μL of CCK-8 was added to the cells and they were incubated for another 4 h. Cell viability was measured at 490 nm in a microplate absorbance reader (Bio-Rad).

### Flow cytometry and Annexin V-FITC/PI staining

Expression of cell surface markers CD34 and CD38 was evaluated by flow cytomerty (FACS Calibur; BD Company) with CD34-PE and CD38-FITC antibodies (BD Company). For the apoptosis assay, cells were transfected with miR-203, miR-NC, si-survivin, or siRNA-NC for 48 h. They were then harvested and10 μL of binding reagent and 2.5 μL Annexin V-FITC (KeyGen BioTech) were added. After 20 min, cells were washed twice with cold PBS and stained with 10 μL PI for 10 min at room temperature. All data were analyzed and calculated using FlowJo software.

### Soft agar clone colony and sphere formation assays

For the soft agar colony formation experiments, LSCs were first treated with miR-203 or miR-NC for 48 h and then seeded at a low density (200 cells/well) in 6-well plates. Cells were cultured for 2 weeks in soft agar until visible colonies appeared. For the spheroid formation assay, 500 single LSCs were isolated and seeded in serum-free stem cell media(Stem Cell Technologies) supplemented with 20 ng/mL epidermal growth factor (Sigma-Aldrich), 10 ng/mL basic fibroblast growth factor (Invitrogen), and 1 × B27 (Life Technologies) in an ultralow attachment plate. Culture media was replenished every 3 days and spheres were counted after 2 weeks.

### Dual-luciferase reporter assays

The survivin and Bmi-1 3′-UTRs containing predicted miR-203 binding sites (both wild-type and mutant) were introduced into pMIR-REPORT plasmid downstream of a luciferase reporter gene. For the luciferase reporter assay, LSCs were seeded in 24-well plates for 24 h, and co-transfected with 50 nmol/L miR-203 or miR-NC, pMIR-REPORT plasmids containing wild-type or mutant survivin or Bmi-1 3′-UTR, and 1 μg *Renilla* luciferase control vector (Promega) as an internal control for normalization of transfection efficiency. The relative luciferase activities were evaluated 48 h later using the Dual Luciferase Reporter Assay Kit (Promega).

### Tumor xenograft model

Four- to five-week old nude mice were randomized into 2 groups and treated with scramble control (miR-NC) or miR-203-infected LSC cells (1 × 10^5^ cells per animal) via subcutaneous transplantation. The whole experiment was performed in accordance with a protocol approved by the Jinan University Institutional Animal Care and Use Committee. The tumor size was measured every 2 days with calipers, and tumor volume was calculated as L × W^2^ × 0.5 (mm^3^) (L indicates length, and W indicates width), until tumors were harvested and weighted after sacrificing the mice.

### Statistical analysis

All statistical analyses were conducted using SPSS 17.0 (SPSS, Chicago, USA) and GraphPad Prism version 5 (GraphPad Software, USA). Unpaired 2-tailed Student’s t test was used to compare differences between treatments for cell viability, the expression levels of miR-203 and its target, and cloning and spheroid-forming efficiencies. All results are presented as the mean ± SD.*P* < 0.05 was considered statistically significant.

## Additional Information

**How to cite this article**: Zhang, Y. *et al.* miR-203 inhibits proliferation and self-renewal of leukemia stem cells by targeting survivin and Bmi-1. *Sci. Rep.* **6**, 19995; doi: 10.1038/srep19995 (2016).

## Supplementary Material

Supplementary Information
