# Supplementary material for: miR-203 inhibits proliferation and self-renewal of leukemia stem cells by targeting survivin and Bmi-1
Source: Sci Rep. 2016 Feb 5;6:19995. doi: 10.1038/srep19995 (PMC4742816; doi:10.1038/srep19995)
Supplement: Supplementary Information [file srep19995-s1.doc]

**miR-203 inhibits proliferation and self-renewal of leukemia stem cells by targeting survivin and Bmi-1**

Yi Zhang^1,2*,7^, Shu-yan Zhou^3*^, Hai-zhao Yan^1^,Dan-dan Xu^1^, Hai-xuan Chen^4^, Xiao-yan Wang^2^, Xiao Wang^2^, Yu-ting Liu^1^, Li Zhang^1^, Sheng Wang^1^, Peng-jun Zhou^5^, Wu-yu Fu^5^, Bi-bo Ruan^5^,Dong-lei Ma^1^, Ying Wang^1^, Qiu-ying Liu^2^, Zhe Ren^2^, Zhong Liu^2^, Rong Zhang^6§^, Yi-fei Wang^1,2§^

^1^College of life science and technology, Jinan University, Guangzhou, 510632, P.R. China

^2^Institute of Biomedicine, Jinan University, Guangzhou, 510632, P.R. China

^3^Department of Pathological Physiology, Wan-nan Medical College,Wuhu, 241000,

 P.R. China

^4^College of medicine, Jinan University, Guangzhou, 510632, P.R. China

^5^Guangdong Pharmaceutical University

^6^Department of Endoscopy, Sun Yat-sen University Cancer Center, State Key Laboratory of Oncology in South China,Collaborative Innovation Center for Cancer Medicine,
Guangzhou, 510632, P.R. China

^7^Section of Otolaryngology, Department of Surgery, Yale School of Medicine

*These authors contributed equally to this work

^§^Corresponding author

Email Address:

Yi Zhang: [scholarzy@163.com](mailto:scholarzy@163.com)

Shu-yan Zhou: [zhoushuyan_xju2005@126.com](mailto:zhoushuyan_xju2005@126.com)

Hai-zhao Yan: yanhaizhao@126.com

Dan-danXu: Junxu98@163.com

Hai-xuan Chen: [chx00520@163.com](mailto:chx00520@163.com)

Xiao-yan Wang: [wangxiaoyan_1016@163.com](mailto:wangxiaoyan_1016@163.com)

Xiao Wang: [wangxiao0719@163.com](mailto:wangxiao0719@163.com)

Yu-ting Liu: [ziziniu@163.com](mailto:ziziniu@163.com)

Li Zhang: [15692002254@163.com](mailto:15692002254@163.com)

Sheng Wang: 18520215232@163.com

Peng-jun Zhou: zhouzhang1990@163.com

Wu-yu Fu: fuwuyu2014@163.com

Bi-boRuan: [15011931237@163.com](mailto:15011931237@163.com)

Dong-lei Ma: donglei131001@163.com

Ying Wang: wy1005swau@aliyun.com

Qiu-ying Liu: qiuying_liu@126.com

ZheRen: rz62@163.com

Zhong Liu: tliuzh@jnu.edu.cn

Rong Zhang: zhangrong@sysucc.org.cn

Yi-fei Wang: [twang-yf@163.com](mailto:twang-yf@163.com)

(0086-021-85220504)

**Table S1. Demographics and clinical characteristics of 50 newly diagnosed AML patients in this study**

| **Characteristics (n)** | **miR-203**  **CD34+ expression, n (%) CD34- expression, n (%)** |
| --- | --- |
| Sex |  |
| Female (29) | 18 (62%) 11 (38%) |
| Male (21) | 7 (33%) 14 (67%) |
| *P*value | 0.157 |
| Age |  |
| ≤40 years old (37) | 23 (62%) 14 (38%) |
| >40 years old (13) | 6 (46%) 7 (54%) |
| *P*value | 0.165 |
| FAB |  |
| M0 (7) | 3 (43%) 4 (57%) |
| M1 (10) | 2 (20%) 8 (80%) |
| M2 (11) | 4 (36%) 7 (64%) |
| M5 (13) | 7 (54%) 6 (46%) |
| M7 (9) | 6 (67%) 3 (33%) |
| *P*value | 0.212 |
| Laboratory analysis (median) |  |
| WBC (10^9^/L) | 24.3 17.2 |
| Platelet (10^9^/L) | 5648 |
| Hemoglobin (g/dL) | 12.89.3 |
| BM blast, % | 4532 |
| PB blast, % | 23 14 |
| *P*value | 0.189 |

Abbreviations: FAB, French-American-Britain subtype; WBC, white blood cell; LDH, lactate dehydrogenase; BM, bone marrow; PB, peripheral blood. *Fisher’s exact test was used.

| **Table S2. Primers for qRT-PCR**   | **Construct Primer sequences (5’-3’)** | | | --- | --- | | Survivin  Bmi-1  FLT3  WT1  ABCG2  GAPDH | F: CACCGCATCTCTACATTCAAGA  R: AAGTCTGGCTCGTTCTCAGTG  F: GGCTCTAATGAAGATAGAGGAG  R: TCACAGTCATTGCTGCTGGGCA  F: GATAGTCACTGAATTCGGTCTTGGA  R: GACATTCTCTGCCACAGAAAACAGC  F: TTACTGGGTGAGGAAATCCA  R: AGATCAACACCCAGTGATGC  F: TCAATCAAAGTGCTTCTTTTTTATG  R: TTGTGGAAGAATCACGTGGC  F: AACGGATTTGGTCGTATTGGG  R: TCGCTCCTGGAAGATGGTGAT |   **Supplemental Figure Legends**  **Supplemental Fig.1 qRT-PCR of miR-203 expression levels**  miR-203 levels were detected after transfection a miR-203 mimic 48h in LSC-enriched  KG-1a and MOLM-13 cell lines and referenced against RNU6B.  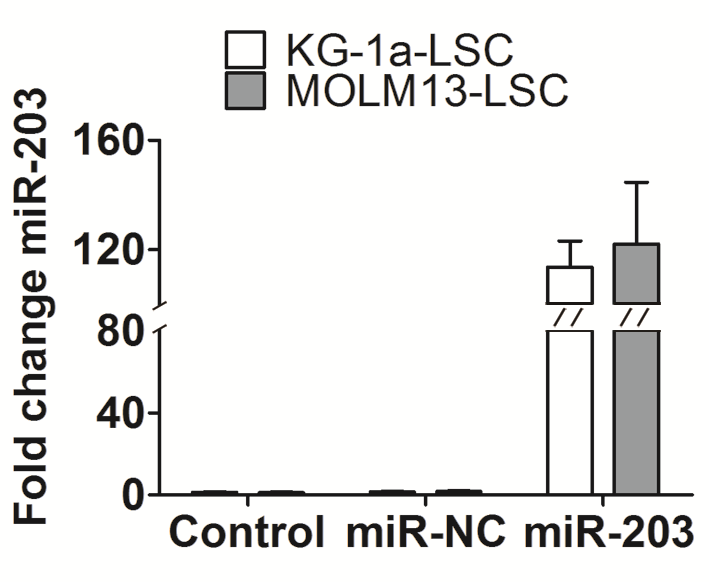 |
| --- | --- | --- | --- | --- |
